# Hemodynamic responses are reduced with aerobic compared with resistance blood flow restriction exercise

**DOI:** 10.14814/phy2.13142

**Published:** 2017-02-09

**Authors:** Anthony K. May, Christopher R. Brandner, Stuart A. Warmington

**Affiliations:** ^1^ Institute for Physical Activity and NutritionSchool of Exercise and Nutrition SciencesDeakin UniversityBurwoodVictoriaAustralia; ^2^ Sport Science DepartmentAspire AcademyDohaQatar

**Keywords:** BFR exercise, hemodynamics, kaatsu, vascular occlusion

## Abstract

The hemodynamics of light‐load exercise with an applied blood‐flow restriction (BFR) have not been extensively compared between light‐intensity, BFR, and high‐intensity forms of both resistance and aerobic exercise in the same participant population. Therefore, the purpose of this study was to use a randomized crossover design to examine the hemodynamic responses to resistance and aerobic BFR exercise in comparison with a common high‐intensity and light‐intensity non‐BFR exercise. On separate occasions participants completed a leg‐press (resistance) or treadmill (aerobic) trial. Each trial comprised a light‐intensity bout (LI) followed by a light‐intensity bout with BFR (80% resting systolic blood pressure (LI+BFR)), then a high‐intensity bout (HI). To characterize the hemodynamic response, measures of cardiac output, stroke volume, heart rate and blood pressure were taken at baseline and exercise for each bout. Exercising hemodynamics for leg‐press LI+BFR most often resembled those for HI and were greater than LI (e.g. for systolic blood pressure LI+BFR = 152 ± 3 mmHg; HI = 153 ± 3; LI = 143 ± 3 *P *<* *0.05). However, exercising hemodynamics for treadmill LI+BFR most often resembled those for LI and were lower than HI (e.g. for systolic pressure LI+BFR = 124 ± 2 mmHg; LI = 123 ± 2; HI = 140 ± 3 *P *<* *0.05). In conclusion, the hemodynamic response for light aerobic (walking) BFR exercise suggests this mode of BFR exercise may be preferential for chronic use to develop muscle size and strength, and other health benefits in certain clinical populations that are contraindicated to heavy‐load resistance exercise.

## Introduction

Undertaking heavy‐load resistance exercise (HLRE) is not often appropriate for ‘at risk’ individuals such as older adults due to the associated substantial elevations in blood pressure (BP), even when only small muscle groups are activated (Macdougall et al. 1985; Miyachi et al. 2004). Similarly, for otherwise healthy adults in injury rehabilitation, maintenance of muscle mass and strength through HLRE is often avoided for some weeks, while training may be directed away from resistance to more aerobic (light) forms of exercise (Wernbom et al. 2008). A relatively novel alternative may be to combine light‐load resistance exercise with an applied blood‐flow restriction (BFR) given BFR training has been shown to induce significant gains in muscle mass and strength (Takarada et al. 2000; Yasuda et al. 2011; Kubo et al. 2006). Interestingly, BFR combined with light‐intensity aerobic exercise such as walking also generates significant gains in muscle mass and strength in adult (Abe et al. 2006) and older adult (Abe et al. 2010) populations. This potential for aerobic exercise to maintain or improve muscle mass and strength suggests aerobic BFR exercise may be an appropriate inclusion when designing exercise programmes for clinical populations, or those with elevated cardiovascular risk, provided that the hemodynamic responses approximate those of light‐intensity exercise without BFR, rather than HLRE. However, to date the hemodynamic responses to both resistance and aerobic BFR exercise have not yet been appropriately characterized within their respective low‐to‐high‐intensity exercise spectra that would seem necessary to inform on the most appropriate modes of BFR exercise for use in various healthy and clinical populations.

While the hemodynamic responses to BFR resistance exercise have previously been compared with similar light‐load non‐BFR resistance exercise as well as HLRE in the same study (Downs et al. 2014; Brandner et al. 2015; Poton and Polito 2016), these comparisons did not examine these exercising responses under typical BFR conditions that use a partial restriction to limb blood flow and a protocol that comprised an initial set of 30 repetitions followed by three sets of 15 repetitions. For example, while resistance exercise to failure in each of three sets demonstrated greater hemodynamic stress compared with HLRE, this may have even under‐represented the hemodynamic response to BFR exercise due to a reduction in the work performed and short timeframe of exercise in the final 2 or 3 sets, the interpretation of which may be complicated by an initial exercise set to failure (Downs et al. 2014). Conversely, examples of complete occlusion of limb blood flow appear to exaggerate the hemodynamic response to BFR exercise (Poton and Polito 2016; Yasuda et al. 2009). In addition, while a valuable examination has been made of the hemodynamics of small muscle groups using BFR of the upper arm (Brandner et al. 2015), unilateral bicep curl exercises are not typically prescribed to achieve systemic health benefits and limit the hemodynamic stress of the exercise when compared with lower body exercises where a greater total mass of muscle is recruited (Macdougall et al. 1985; Lewis et al. 1985).

Moreover, a direct comparison of the hemodynamic responses of resistance and aerobic BFR exercise has also not been made against a high‐intensity equivalent in the same study population. However, investigations of the hemodynamic responses to resistance and aerobic BFR exercise training of the legs compared with similar light‐intensity non‐BFR exercise have been well characterized (Renzi et al. 2010; Staunton et al. 2015; Takano et al. 2005; Ozaki et al. 2010; Iida et al. 2007). During aerobic BFR exercise BP appears to rise to levels greater than similar light‐intensity non‐BFR exercise (Renzi et al. 2010; Staunton et al. 2015). Likewise, during light‐load BFR resistance exercise, the increase in BP appears greater than a non‐BFR control (Takano et al. 2005). In contrast, stroke volume (SV) can be lower during BFR exercise when compared with non‐BFR control exercise (Takano et al. 2005; Renzi et al. 2010; Ozaki et al. 2010). This is most likely due to the applied BFR limiting venous return, while additionally increasing cardiac after‐load (Takano et al. 2005; Renzi et al. 2010; Iida et al. 2007). As such, a subsequent compensatory increase in HR appears to maintain cardiac output ($\dot{Q}$) to levels similar to non‐BFR control exercise (Renzi et al. 2010; Takano et al. 2005; Ozaki et al. 2010).

While we have previously demonstrated BFR exercise to induce a greater hemodynamic stress compared with equal‐intensity exercise without BFR, this was with arm exercise (Brandner et al. 2015), and leg exercises that did not compare against a high‐intensity mode of exercise (Staunton et al. 2015). Therefore, it is important to characterize how this stress may compare with a representative high‐intensity form of exercise, especially considering that BFR exercise has been proposed as a potential alternative to HLRE in clinical populations (Takarada et al. 2000). Consequently, this study aimed to characterize the hemodynamic responses ($\dot{Q}$, SV, HR, BP) to resistance and aerobic BFR exercise in comparison with a typical high‐intensity and light‐intensity non‐BFR exercise as representatives of the two extreme ends of the spectrum for these two modes of exercise. It was hypothesized that for both resistance and aerobic BFR exercise that the magnitude of the hemodynamic responses would be greater than for non‐BFR light‐intensity exercise but lower than high‐intensity exercise.

## Materials and Methods

### Subjects

Fourteen (*n* = 14) recreationally active young men were recruited to participate in this study (Table 1). All participants were untrained in resistance exercise for the previous 12 months and underwent a medical/exercise pre‐screening. Participants were otherwise healthy without previously being diagnosed with cardiovascular disease. Potential participants were excluded if they had any known musculoskeletal or neurological impairment that may have affected their capacity to undertake the exercise and testing requirements of the study, or if they were taking prescribed medication for blood pressure control with or without a history of abnormal blood pressure. All participants had not previously identified as being smokers over their lifetime. Informed consent was obtained from all participants included in the study. This study was approved by the Human Ethics Advisory Group, Deakin University and was performed in accordance with the ethical standards as laid down in the 1964 Declaration of Helsinki and its later amendments or comparable ethical standards.

**Table 1:** Participant characteristics and exercise values

Subject anthropometry	
Age (years)	22 ± 1
APHR_max_ (beats·min^−1^)	192 ± 1
Height (m)	1.79 ± 0.06
Body mass (kg)	74.9 ± 12.0
LP trial characteristics	
1 RM (kg)	287 ± 41
80% 1 RM (kg)	229 ± 33
20% 1 RM (kg)	57 ± 8
Resting sBP (mmHg)	125 ± 8
BFR cuff pressure (mmHg)	100 ± 6
TM trial characteristics	
*V̇*O_2_max (L·min^−1^)	3.7 ± 0.5
*V̇*O_2_max (mL·kg^−1^·min^−1^)	50.3 ± 6.7
Resting sBP (mmHg)	118 ± 9
Estimated thigh sBP (mmHg)	154 ± 10
BFR cuff pressure (mmHg)	123 ± 8

Data are mean ± SD.

### Experimental design

This study followed a balanced randomized crossover design. Participants were required to visit the laboratory on three occasions separated by at least 7 days. At the first visit participants completed a familiarization session, while for subsequent visits participants were randomly allocated to perform either an aerobic treadmill exercise trial (TM) or leg‐press trial (LP), completing the alternate trial at the final visit.

### Familiarization session

The familiarization session comprised an initial determination of maximal oxygen uptake (*V̇*O_2_ max) via a graded exercise test on a treadmill. Following this test participants were instructed and practiced in the breathing patterns required for the measurement of $\dot{Q}$ via rebreathing during the trials. A familiarization with BFR was then undertaken but while performing arm bicep curl exercise to avoid any residual fatigue that may affect the final test that was to determine maximal 45° double leg‐press strength (one repetition maximum; 1‐RM).

#### Graded treadmill test

All treadmill exercise was conducted using a Quinton Q65 Stress Test Treadmill (Quinton, Seattle). For the incremental test, initial treadmill velocity was 8 km·h^−1^. Velocity then increased by 1.5 km·h^−1^ every 3 min until 11 km·h^−1^. After this, treadmill velocity increased by 1 km·h^−1^ each minute until 13 km·h^−1^, and then by 0.5 km·h^−1^ each minute until volitional exhaustion. Expired gasses were analyzed throughout the test on a breath‐by‐breath basis to estimate *V̇*O_2_ max (Innocor DK‐5260, Innovision, Odense, Denmark).

#### 45° double leg‐press 1‐RM

All leg‐press exercise was double leg and undertaken using a standard 45° leg‐press. A determination of 1‐RM was conducted according to methods described previously (Gordon 2010). Briefly, participants began with a specific warm‐up set of eight repetitions at ~125 kg (50% of estimated 1‐RM). Repetitions were defined as a controlled movement with good posture, starting at full knee and hip extension, lowering to 90° knee flexion then returning to full leg extension. This was followed by a single repetition at ~175 kg (70% of estimated 1‐RM). Single repetition lifts were then conducted with progressively heavier loads until failure. Load at failure was defined as the final load that could be successfully lifted with proper technique where an additional 5 kg could not be successfully lifted. Rest intervals between 1‐RM attempts were dependent on participant readiness but ranged from 3 to 5 min, while not more than four single lifts were completed during any test.

### Experimental trials

**Figure 1:**
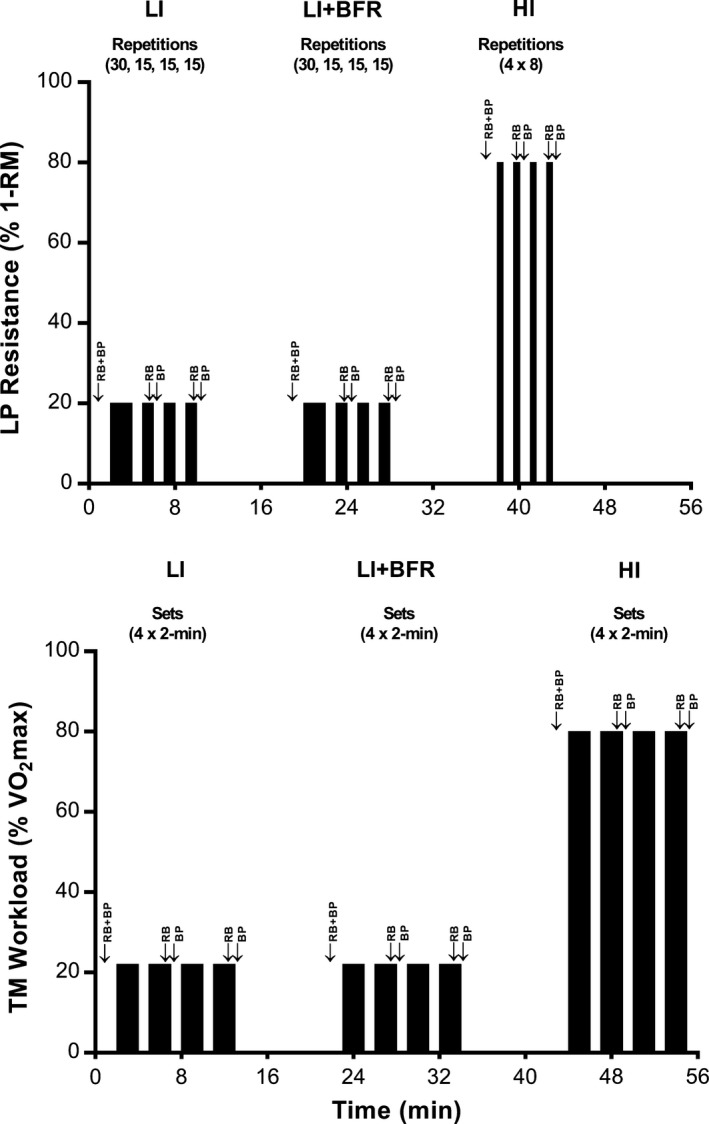
Timelines for leg‐press and treadmill experimental trials. Timing of measurements of $\dot{Q}$, SV and HR are indicated with RB (rebreathing), while blood pressure measurements are indicated with BP.

Each experimental trial (TM and LP) comprised three bouts of exercise each separated by a 10‐min rest period (Fig. 1). Each bout comprised four sets separated by 1‐min rest periods. The initial bout was of light‐intensity exercise (LI), the second bout was also light‐intensity exercise but with an applied BFR (LI+BFR), while the third bout was of high‐intensity exercise without BFR (HI). For the LP trial, the LI and LI+BFR bouts comprised a standard BFR exercise protocol with one set of 30 repetitions followed by three sets of 15 repetitions performed at 20% 1‐RM (Fry et al. 2010; Fujita et al. 2007), while HI comprised four sets of eight repetitions at 80% 1‐RM as a load representative of more traditional resistance training. Repetitions were performed at a fixed cadence timed by a metronome (2‐sec concentric; 2‐sec eccentric). For the TM trial, all sets were 2‐min in duration. LI and LI+BFR required participants to walk at a velocity of 4 km·h^−1^ as used previously (Sugawara et al. 2015; Renzi et al. 2010). HI required participants to run at a velocity equivalent to 80% *V̇*O_2_ max as a workload representative of a high submaximal intensity.

#### Blood‐flow restriction

For all LI+BFR bouts, BFR was applied using an automatic tourniquet system (ATS 3000, Zimmer Inc., OH) connected to 10.5 cm wide cuffs placed as high as possible on each thigh. BFR was applied for the duration of the bout (i.e. throughout all sets and rest periods) and released at the conclusion of the bout immediately prior to the 10‐min rest period. For the TM trial, the applied cuff pressure was individualized to 80% of the baseline resting systolic blood pressure (sBP) at the level of the BFR cuff, estimated according to the following equation (Egaña et al. 2006):

$$sBP_{\mathrm{thigh}}=sBP_{\mathrm{brachial}}+\left(\left(g\times d\times h\right)\times 0.0075\right)$$

where *sBP*_*thigh*_ **= estimated sBP (mmHg) at the level of the BFR cuff; *sBP*_*brachial*_ = standard sBP measured at the level of the brachial artery (mmHg); *g * = acceleration due to gravity (9.8 m·sec^−2^); *d * = blood density (1060 kg·m^−2^); and *h * = vertical distance between the midpoints of the brachial sphygmomanometer cuff and the BFR cuff (m).

For the LP trial, the vertical distance between the brachial sphygmomanometer and thigh BFR cuffs was negligible when in the leg‐press position. Therefore, the applied BFR pressure was equal to 80% of the measured baseline resting brachial sBP (Table 1).

### Measurements

#### Hemodynamic parameters

All hemodynamic measures, ($\dot{Q}$, SV, HR, BP) were collected at baseline prior to the commencement of LI and after 8 min of the 10‐min rest period before commencement of LI+BFR and HI. During bouts, $\dot{Q}$ and HR were measured over the final 3–5 repetitions of exercise sets 2 and 4 while an automated determination of BP was initiated immediately after completion of sets 2 and 4 of each bout and was complete within 30‐sec.

The measurement of $\dot{Q}$ was made using an online metabolic system (Innocor DK‐5260, Innovision, Odense, Denmark) via a standard inert gas re‐breathing technique as previously described (Fontana et al. 2009; Jakovljevic et al. 2008). A specific breathing duration was not prescribed during TM but LP required participants to breathe at a duration of 2‐sec inhalation and 2‐sec exhalation. This allowed for breathing patterns to be in time with repetitions in an attempt to minimize any effect of a change in abdominal pressure on both $\dot{Q}$ and blood pressure measurements. HR was obtained using a standard chest strap and wrist unit (RS800CX, Polar Electro, Kemple, Finland), with SV estimated as the quotient of $\dot{Q}$ and HR. Age‐predicted maximum HR (APHR_max_) was estimated according to the formula [206.9–(0.67 × age)] (Gellish et al. 2007).

Brachial artery blood pressures (sBP; diastolic blood pressure (dBP); mean arterial pressure (MAP)) were measured via an automated brachial sphygmomanometer controlled by the online metabolic system (SunTech Medical, Guanlan, Shenzhen).

#### Ratings of perceived exertion

At the completion of each bout (LI, LI+BFR, HI), participants were required to provide a rating of perceived exertion (RPE) on a standard 15 point Borg scale ranging from 6 (No exertion at all) to 20 (Maximal exertion) (Borg 1970).

### Data presentation and statistical analyses

All hemodynamic data were initially analysed with a two‐factor repeated measures analysis of variance (ANOVA) for BOUT (LI, LI+BFR, HI) and TIME (baseline, set 2, set 4). No differences were observed for any parameter for set 2 versus set 4 and so these data were averaged to instead provide a value for EXERCISE that was re‐examined using a similar ANOVA design. This was followed by a Tukey‐Kramer post hoc test to identify where specific differences occurred. RPE and pre‐exercise hemodynamic variables were similarly analysed with a two‐factor repeated measures ANOVA for TRIAL (LP and TM) and BOUT (LI, LI+BFR, HI). Unless otherwise stated all data are presented as mean±SEM. Differences were considered significant at a level of *P *<* *0.05. Statistics were computed using NCSS (v2007, NCSS LLC, Utah).

## Results

Characteristics of participants and of each exercise trial are displayed in Table 1. For LP, average load (mean ± SD) was 57 ± 8 kg (LI, LI + BFR), and 229 ± 33 kg (HI). For TM, walking speed was 4 km·h^−1^ for LI and LI+BFR, and this was equivalent to 22 ± 5% *V̇*O_2_ max and 22 ± 4% *V̇*O_2_ max, respectively. For HI, average running speed was 11.5 ± 2.4 km·h^−1^ (80% *V̇*O_2_ max).

Prior to exercise, postural differences between LP and TM trials showed $\dot{Q}$, SV and sBP to be greater in LP when compared with TM (*P *<* *0.05) (Table 2). In contrast, HR measures were lower prior to LP when compared with TM (*P *<* *0.05). Pre‐exercise dBP (*P *=* *0.56) and MAP (*P *=* *0.36) were not different between LP and TM.

**Table 2:** Physiological values during exercise trials

	LI		LI+BFR		HI	
	Baseline	Exercise	Baseline	Exercise	Baseline	Exercise
LP trial						
sBP (mmHg)	125 ± 2	143 ± 3^a^^,^ b	127 ± 2	152 ± 3^a^	127 ± 2	153 ± 3^a^
dBP (mmHg)	67 ± 2	75 ± 1^a^	68 ± 2	85 ± 2^a^^,^ c	65 ± 2	71 ± 2
MAP (mmHg)	86 ± 2	97 ± 2^a^	87 ± 1	107 ± 2^a^^,^ c	86 ± 2	98 ± 2^a^
HR (beats·min^−1^)	65 ± 3	109 ± 4^a^	71 ± 4^d^	116 ± 4^a^^,^ c	76 ± 4^d^	138 ± 5^a^^,^ c
HR (% APHR_max_)	34 ± 2	55 ± 3^a^	37 ± 2^d^	58 ± 3^a^^,^ c	39 ± 2^d^	72 ± 2^a^^,^ c
SV (mL)	112 ± 9	109 ± 5	120 ± 8	102 ± 5^a^	124 ± 8	94 ± 4^a^^,^ e
$\dot{Q}$ (L·min−1)	7.2 ± 0.5	11.5 ± 0.4^a^^,^ b	8.3 ± 0.6	11.5 ± 0.3^a^^,^ b	9.1 ± 0.6	12.6 ± 0.5^a^
TM trial						
sBP (mmHg)	118 ± 2	123 ± 2	118 ± 4	124 ± 2	117 ± 2	140 ± 3^a^^,^ c
dBP (mmHg)	68 ± 2	72 ± 2^a^	68 ± 2	76 ± 2^a^^,^ b	67 ± 2	68 ± 2^a^
MAP (mmHg)	85 ± 2	89 ± 2^a^	84 ± 2	92 ± 2^a^	83 ± 1	91 ± 2^a^
HR (beats·min^−1^)	68 ± 3	88 ± 3^a^	71 ± 3	92 ± 3^a^	73 ± 3	157 ± 3^a^^,^ c
HR (% APHR_max_)	35 ± 2	46 ± 1^a^	37 ± 2	48 ± 1^a^	38 ± 2^e^	79 ± 3^a^^,^ c
SV (mL)	75 ± 5	109 ± 5^a^	72 ± 6	96 ± 4^a^	70 ± 5	119 ± 5^a^^,^ f
$\dot{Q}$ (L·min−1)	5.1 ± 0.4	9.5 ± 0.3^a^	5.1 ± 0.3	8.6 ± 0.2^a^	5.0 ± 0.3	18.5 ± 0.8^a^^,^ c

Data are Mean ± SEM. BFR, blood‐flow restriction; LI, light‐intensity; HI, high‐intensity; LP, leg‐press trial; TM, treadmill trial.

a Different to Baseline (*P *<* *0.05).

b Main effect for BOUT versus HI (*P *<* *0.05).

c Different to EXERCISE in all other BOUTS (*P *<* *0.05).

d Different to Baseline in all other BOUTS (*P *<* *0.05).

e Different to LI only (*P *<* *0.05).

f Different to LI+BFR only (*P *<* *0.05).

### Leg‐press trial

For LP, hemodynamic responses to each bout (LI, LI+BFR, HI) are presented in Table 2. sBP, dBP, and MAP increased from baseline with each exercise bout, except dBP in HI. The response of dBP and MAP to exercise was greater for LI+BFR compared with both LI and HI. For sBP there was a main effect for BOUT such that HI was greater than LI, yet despite this sBP values with exercise were not different between bouts.

HR increased from baseline to exercise in all bouts and was greater during exercise in LI+BFR (116 ± 4 beats·min^−1^) compared with LI (109 ± 4 beats·min^−1^) but lower when compared with HI (138 ± 5 beats·min^−1^) (*P *<* *0.05). $\dot{Q}$ also increased from baseline to exercise in all bouts. However, $\dot{Q}$ was not different during exercise between bouts despite a main effect for Bout such that HI was greater than both LI and LI+BFR. SV decreased from baseline to exercise in LI+BFR (102 ± 5 mL) and HI (94 ± 4 mL) but remained unchanged in LI, and was significantly lower during exercise in HI compared with LI (109 ± 5 mL) (*P *<* *0.05).

### Treadmill trial

For TM, hemodynamic responses to each bout are presented in Table 2. All variables were similar at baseline between bouts and increased with exercise, except for sBP in LI and LI+BFR. This elevation was greater with exercise in HI than both LI and LI+BFR for sBP, HR, and $\dot{Q}$,while SV was greater during exercise in HI when compared with LI+BFR only. No hemodynamic variable was different between LI and LI+BFR.

### Ratings of perceived exertion

**Figure 2:**
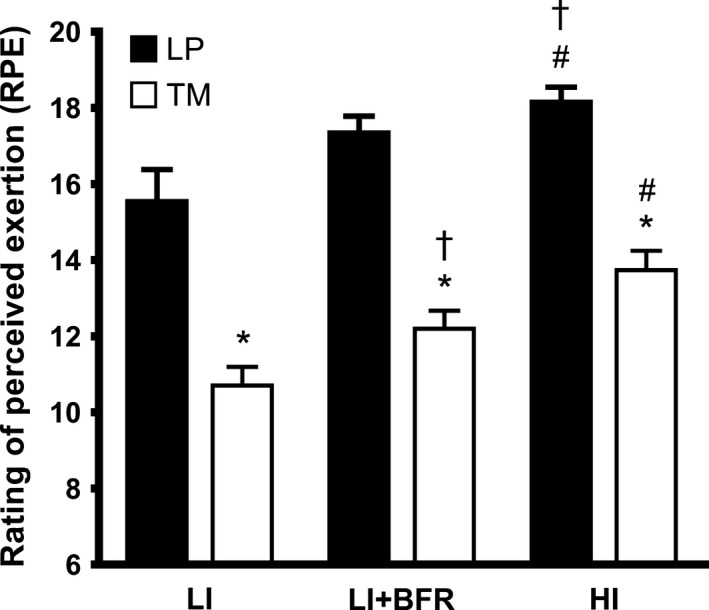
Ratings of perceived exertion (Borg scale) within LP and TM trials. Data are Mean ± SEM. *denotes different to LP (*P *<* *0.05), ^†^denotes different to LI (*P *<* *0.05), ^#^denotes different to LI and LI+BFR (*P *<* *0.05).

Respective RPE scores for both LP and TM increased progressively from LI (15.6 ± 0.8 and 10.8 ± 0.4), to LI+BFR (17.4 ± 0.4 and 12.3 ± 0.4), to HI (18.2 ± 0.3 and 13.8 ± 0.5) (Fig. 2). However, for each bout RPE scores were all greater in LP than TM.

## Discussion

The major findings of the present study were (1) that light‐load resistance exercise combined with BFR increased the hemodynamic response when compared with a similar light‐load resistance exercise without an applied BFR, and for BP (MAP) this was even greater than for HLRE; (2) that light‐intensity aerobic exercise combined with BFR induced hemodynamic responses that were similar to those of an equal‐intensity non‐BFR aerobic exercise, but were typically lower than a high‐intensity aerobic exercise; and (3) that aerobic BFR exercise was perceived to be easier to perform than BFR resistance exercise under the conditions and selected loads of the present study.

### Leg‐press

For the leg‐press trial we demonstrated the nature of the HR response to exercise to be progressively greater from the LI to LI+BFR to HI bouts (Table 2). However, while HR may be a ready measure for practitioners to make some judgment about the extent of the hemodynamic response to exercise (especially for BFR exercise), it is important to note that this HR response appears to compensate for the progressive reduction in SV from HI to LI+BFR to LI observed in the present study, and previously (Brandner et al. 2015; Poton and Polito 2016). Together, these HR and SV data showed exercising $\dot{Q}$ to be similar between all bouts, which is common in studies that have only compared BFR resistance exercise with an equivalent light‐intensity exercise (Takano et al. 2005; Staunton et al. 2015). In contrast, very few studies demonstrate cardiac measures and BP measures of hemodynamics for LI, LI+BFR and HI in the same study population (Downs et al. 2014; Brandner et al. 2015; Poton and Polito 2016). Hence, the present study is novel by being the first to compare these three exercise modes while employing a standard lower body BFR resistance exercise (4 sets; 30, 15, 15, 15 reps), even though others have examined these lower‐body hemodynamic responses to repetitions until muscle failure (Downs et al. 2014), or with less repetitions during full blood flow occlusion (Poton and Polito 2016). It is also important to note that the 45° leg‐press exercise used in the present study provides an opportunity to observe a postural effect on SV in LI such that baseline SV was most likely elevated compared with a more standard exercising posture where the legs are below the level of the heart and the hips (Table 2). As a consequence, exercising SV remained unchanged from baseline in LI, whereas HR increased substantially. Despite this effect of posture, the application of BFR demonstrates a significant reduction in exercising SV in the LI+BFR bout that may stem from a restriction to venous return (Iida et al. 2007). Interestingly, this reduction in SV appears strongest in HI and as such it may suggest that HLRE may induce a significant restriction of flow through the vasculature of active muscle during the contraction phase due to high intramuscular pressures (Pereira et al. 2007). Consequently, while there is currently no empirical data, it is tempting to speculate that this may be linked to a mechanism by which BFR resistance exercise increases muscle size and strength when used chronically across a training program, although this requires further investigation.

In contrast with the cardiac measures of hemodynamics, the BP responses to leg‐press exercise in the present study follow a different pattern that was not progressive from LI to LI+BFR to HI. Instead, LI+BFR demonstrated the greatest dBP response, and while sBP was similar to HI, this translated into a significantly greater MAP for LI+BFR versus both LI and HI. Again, these data are similar to previous comparisons of BFR resistance exercise with equal‐intensity non‐BFR resistance exercise (Staunton et al. 2015; Vieira et al. 2013; Takano et al. 2005), while making a novel contribution to show that the BP response to lower body BFR resistance exercise may be at least equivalent to that for traditional HLRE. However, the elevation in MAP with leg‐press BFR exercise was manifest through an elevation in dBP, not sBP. While an elevated dBP is a concern, 84 ± 2 mmHg is not clinically abnormal and in terms of the risk of an acute cardiovascular event a spike in sBP would certainly be of more concern. While other factors such as muscle group (smaller versus larger/multiple muscles) (Brandner et al. 2015), and posture (Staunton et al. 2015) have been suggested to likely influence the magnitude of the hemodynamic response to BFR exercise, it is typically expected that the hemodynamic response is greater than for non‐BFR exercise but lower than for HLRE (Poton and Polito 2016; Brandner et al. 2015). As such, this should still lead to caution for the prescription of BFR resistance exercise for some populations, in particular those at risk of a cardiovascular event.

### Treadmill

The hemodynamic responses to high‐intensity treadmill running (HI) were significantly greater than both treadmill walking (LI) and walking with an applied BFR (LI+BFR). For blood pressure responses, this is the first investigation to directly compare low‐intensity BFR walking against running at a typical high‐intensity and showed sBP to be significantly greater during HI (unlike the LP trial). This suggests that the application of BFR to light‐intensity aerobic exercise does not result in a BP response like that of intense aerobic activity.

The similar hemodynamic responses between LI and LI+BFR are in contrast to previous reports that observed increased blood pressures with aerobic BFR exercise to levels greater than an equal‐intensity control exercise bout, combined with a greater HR and reduced SV while maintaining $\dot{Q}$ (Renzi et al. 2010; Staunton et al. 2015; Sugawara et al. 2015; Ozaki et al. 2010). Typically, such different responses to BFR exercise between studies may be attributed to factors associated with the application of BFR such as cuff width and cuff pressure (Loenneke et al. 2012a), and the exercise type (Staunton et al. 2015), combined with individual factors such as limb circumference (Loenneke et al. 2012a). Indeed, the present study methodology is very similar to another recent study from our laboratory that showed greater HR and blood pressures with BFR walking exercise compared with a control walking exercise (Staunton et al. 2015). Although both studies used the same BFR cuffs, this difference in hemodynamic stress may be a result of slight differences in the method for applying an individualized restriction pressure being 80% resting sBP (mean ± SD; 123 ± 8 mmHg) in the present study versus 60% of limb occlusion pressure (LOP) (126 ± 16) in the prior study. However, with these methods resulting in similar mean absolute restriction pressures between these two studies, the greater variation associated with the LOP method suggests a more specific individualization to the assignment of restriction pressures that accounts for more factors that affect limb blood flow in an individual that are not accounted for when using a product of sBP. In addition, while both investigations used an identical BFR walking protocol the present study reported a lower pre‐exercise resting HR (68 ± 3 beats·min^−1^ vs. 76 ± 5 beats·min^−1^) and lower resting percentage of APHR_max_ (35 ± 2% APHR_max_ vs. 40 ± 2% APHR_max_) (Staunton et al. 2015) with the average *V̇*O_2_ max of participants in the present study being likely greater, and in the 75th percentile for this age group (50.3 ± 6.7 mL·kg^−1^·min^−1^) (Gordon 2010). Taken together, this suggests that aerobic fitness may be an important factor (among others) for consideration when prescribing aerobic BFR exercise for training or rehabilitation.

Indeed, given that the magnitude of the hemodynamic response in LP was typically greater than in TM, the present study lends some support to aerobic BFR exercise being preferential compared with BFR resistance exercise when undertaken chronically to develop muscle size and strength, in particular when the hemodynamic response is a factor for consideration when selecting the mode to be used for exercise training (Loenneke et al. 2012b). Certainly, aerobic BFR walking exercise conferred a reduced hemodynamic stress in comparison with high‐intensity running (TM in Table 2), whereas for BFR resistance exercise the hemodynamic response appeared at least equivalent, if not greater, to a high‐intensity resistance exercise (LP in Table 2). While this difference in the hemodynamic response to resistance and aerobic BFR exercise is likely driven by a differential degree of motor unit recruitment for the same relative intensity on the spectrum of intensities for each mode of exercise, without any detailed assessment of autonomic function there is no evidence in our data to suggest any differential regulation of the hemodynamic response to aerobic BFR exercise, but perhaps some evidence for BFR resistance exercise. Given the level of HR achieved (~90–110 beats·min^−1^), our data indicate that the rise in hemodynamic responses to BFR exercise at 20% relative intensity are highly dependent on parasympathetic withdrawal in addition to other factors such as vascular compression and the skeletal muscle pump, posture and cuff pressure, all of which require further investigation. However, for BFR resistance exercise (i.e. LP), the HR response was slightly greater than this threshold (116 ± 4) with dBP and MAP also being marginally elevated when compared with LI. Despite a similar sBP and $\dot{Q}$ between LI and LI+BFR, this may suggest a slightly greater degree of sympathetic input. Again, this requires further investigation.

The suggestion that aerobic BFR exercise may be preferential is also supported by the RPE data (Fig. 2) that was significantly lower for all TM bouts (LI, LI+BFR, HI) when compared with the LP bouts. Although LI+BFR was perceived to be less difficult to perform than HI during both LP and TM, the significantly lower RPE for aerobic BFR walking exercise in TM compared with that for light‐intensity BFR resistance exercise in LP suggests the use of aerobic BFR training over resistance exercise (HLRE or light‐load BFR) as a less demanding method to develop muscle strength and mass, and one that would likely improve compliance across a training program. Notwithstanding that the muscle adaptations with BFR resistance exercise are likely greater (Loenneke et al. 2012b).

### Limitations

One limitation within the present study was the ordered prescription of the bouts undertaken within each trial; that is, all bouts were completed in the order LI then LI+BFR then HI. This was chosen to minimize the duration of the trial through minimizing the rest periods between bouts and, therefore, the commitment of participants. As such, HI was always completed last. As a result, for LP there was the progressive increase in the baseline HR (and HR as % of APHR_max_) for each successive bout (i.e. from LI to LI+BFR to HI ‐ Table 2). For TM this was only evident for HR expressed as %APHR_max_, which was greater at baseline in HI compared with LI. This suggests that for both LP and TM the 10‐min rest periods between bouts were almost, but perhaps not quite, sufficient. A subsequent analysis of the change in HR from rest to exercise for LP (data not shown) indicates that the greater exercising HR in LI+BFR in comparison with LI, may indeed be a result of this progressive rise in baseline HR prior to the start of each successive bout, especially given the mean increase in HR from rest to exercise in both LI and LI+BFR was very similar (~45 beats·min^−1^). However, it is important to note that all other hemodynamic variables returned to baseline within the 10‐min recovery period, and so the impact of this elevated HR is expected to be minimal when characterizing the overall hemodynamic response.

Lastly, it is likely that the measurements taken during the LI and LI+BFR bouts for TM were at, or very close to, steady state. However, given the 2 min duration for all sets during all treadmill bouts, measurements taken during HI may have been underestimated if a steady state was not achieved. While there is no current data on the kinetics of cardiopulmonary responses with BFR exercise (e.g. HR or $\dot{V}O_{2}$), given the ‘standard’ prescription of short aerobic bouts (~2 min duration) when undertaking aerobic BFR exercise, it would seem something pertinent to investigate with future research.

## Conclusion

The present study demonstrated that hemodynamic measures with BFR resistance exercise were typically similar to HLRE but greater than aerobic BFR walking exercise, which were similar to a non‐BFR control walking exercise. In addition, lower RPE scores were observed for aerobic BFR exercise compared with BFR resistance exercise. This would suggest that if restricted hemodynamic responses were a necessary feature of exercise prescription to induce gains in muscle size and strength, such as for certain clinical populations, then aerobic BFR exercise may be a more desirable prescription in comparison with high‐intensity exercises or BFR resistance exercise. However, under these circumstances while the RPE scores suggest that exercise/training compliance may be greater for aerobic BFR exercise, the likely muscle adaptations may not be as great as those for BFR resistance exercise. However, this has not been directly tested and requires further investigation.

## Conflict of Interest

The authors declare that they have no conflict of interest.
